# *Arabidopsis* R-SNARE Proteins VAMP721 and VAMP722 Are Required for Cell Plate Formation

**DOI:** 10.1371/journal.pone.0026129

**Published:** 2011-10-11

**Authors:** Liang Zhang, Haiyan Zhang, Peng Liu, Huaiqing Hao, Jing Bo Jin, Jinxing Lin

**Affiliations:** 1 Key Laboratory of Plant Molecular Physiology, Institute of Botany, Chinese Academy of Sciences, Beijing, China; 2 Graduate School of Chinese Academy of Sciences, Beijing, China; 3 State Key Laboratory of Plant Genomics, Institute of Microbiology, Chinese Academy of Sciences, Beijing, China; Purdue University, United States of America

## Abstract

**Background:**

Cell plate formation during plant cytokinesis is facilitated by SNARE complex-mediated vesicle fusion at the cell-division plane. However, our knowledge regarding R-SNARE components of membrane fusion machinery for cell plate formation remains quite limited.

**Methodology/Principal Findings:**

We report the *in vivo* function of *Arabidopsis* VAMP721 and VAMP722, two closely sequence-related R-SNAREs, in cell plate formation. Double homozygous *vamp721vamp722* mutant seedlings showed lethal dwarf phenotypes and were characterized by rudimentary roots, cotyledons and hypocotyls. Furthermore, cell wall stubs and incomplete cytokinesis were frequently observed in *vamp721vamp722* seedlings. Confocal images revealed that green fluorescent protein-tagged VAMP721 and VAMP722 were preferentially localized to the expanding cell plates in dividing cells. Drug treatments and co-localization analyses demonstrated that punctuate organelles labeled with VAMP721 and VAMP722 represented early endosomes overlapped with VHA-a1-labeled TGN, which were distinct from Golgi stacks and prevacuolar compartments. In addition, protein traffic to the plasma membrane, but not to the vacuole, was severely disrupted in v*amp721vamp722* seedlings by subcellular localization of marker proteins.

**Conclusion/Significance:**

These observations suggest that VAMP721 and VAMP722 are involved in secretory trafficking to the plasma membrane via TGN/early endosomal compartment, which contributes substantially to cell plate formation during plant cytokinesis.

## Introduction

Plant cytokinesis is characterized by deposition of cell wall material at the division plane [1], [2]. This process depends on targeted secretion along the phragmoplast, where homotypic fusion of Golgi-derived vesicles gives rise to the cell plate [3]. After the cell plate eventually fuses with the parental plasma membrane, two individual cells are separated by a new cell wall [4], [5]. The maturation of the cell plate to a rigid cell wall is activated by vesicle fusion, a process mediated by the soluble N-ethyl-maleimide sensitive factor attachment protein receptor (SNARE) complex [6], [7], [8]. During this process, t-SNARE on the target membrane and v-SNARE on the transport vesicle membrane assemble into the functional SNARE complex with a tight cluster of four coiled-coil helices called SNARE motifs. Based on the conserved amino acids in SNARE mortif, SNARE proteins can be classified into four groups: Qa-, Qb-, Qc- (t-SNAREs), and R-SNAREs (v-SNAREs) [9], [10]. Within the animal and fungi lineage, R-SNAREs can be also subdivided into short vesicle-associated membrane proteins (VAMPs) or brevins and long VAMPs or longins. Longin proteins have a conserved N-terminal domain that contains a profiling related fold, while brevin proteins lack this domain [11]. However, only longin type R-SNAREs exist in plants. The *Arabidopsis* genome encodes two Sec22-like, two Ykt6-like and 11 VAMP7-like longin R-SNAREs [12]. The VAMP7-like proteins in green plants consist of two major groups: VAMP71 and VAMP72 groups. The VAMP72 group appears to be specific to the green plant lineage and likely represents the R-SNARE components for secretion [13]. Recently, the biological functions of R-SNAREs during plant growth have received considerable attention.

Current knowledge of membrane fusion machinery at the division plane is derived mainly from several cell plate-localized t-SNARE proteins in *Arabidopsis*. In a forward genetic screen for mutations affecting body organization, *Arabidopsis* KNOLLE was identified as the cytokinesis-specific t-SNARE involved in cell plate formation [14], [15]. An interactor of KN, t-SNARE SNAP33, was identified from a yeast two-hybrid screen. Plants carrying mutations in the *SNAP33* gene display cytokinetic defects [16]. Another distinct membrane fusion pathway for cell plate formation involving t-SNARE protein SYP31 and AAA-ATPase AtCDC48 has been proposed, as AtCDC48 specifically interacts with SYP31 but not with KNOLLE *in vitro*-binding assay in spite of the colocalization at cell-division plane between SYP31 or AtCDC48 and KNOLLE [17]. To date, only NPSN11, one R-SNARE candidate for cell-plate membrane fusion machinery, has been speculated in terms of its cellular localization on the cell plate in dividing cells and its ability to interact with KN; however, the T-DNA insertion lines of the *NPSN11* gene developed normally as the wild-type plants [18]. Therefore, evidence for the function of R-SNARE proteins in plant cytokinesis is still insufficient.

In this study, we investigated the function of the R-SNARE proteins VAMP721 and VAMP722 in cell plate formation by analyzing mutant phenotypes and fluorescence localization. We found that *vamp721vamp722* double mutant seedlings resulted in multiple cytokinesis-defective phenotypes, leading to severe dwarf growth. Fluorescence targeting revealed that VAMP721 and VAMP722 were localized to the cell plate in mitotic cells. Moreover, we demonstrated that cytoplasmic VAMP721 and VAMP722 compartments represented the trans-Golgi network (TGN)/early endosomal compartment, which was implicated in cell plate formation. Importantly, *vamp721vamp722* double mutants suppressed the secretion of plasma membrane (PM) proteins. Taken together, these findings suggest that VAMP721 and VAMP722 activities are required for secretory trafficking from TGN to the cell plate in dividing cells and the plasma membrane, extending our knowledge about R-SNARE components for SNARE complex-mediated cell plate membrane fusion and specialized TGN function during plant cytokinesis.

## Results

### *vamp721vamp722* mutations result in seedling lethality

**Figure 1:**
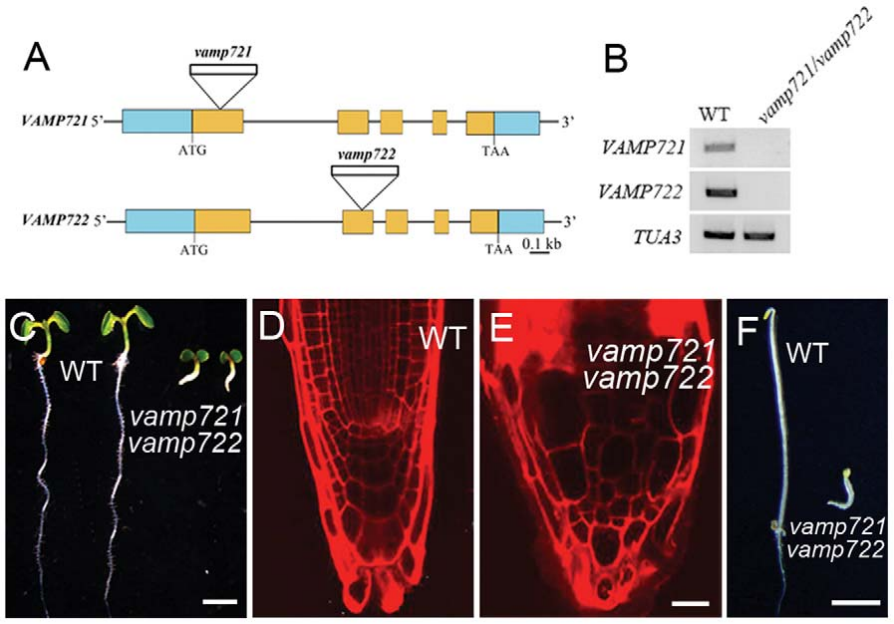
Characterization of *vamp721vamp722* mutants. (A) Schematic structures of *VAMP721* and *VAMP722* genes. Orange boxes represent exons and blue boxes represent UTRs. The sites of T-DNA insertions are indicated. (B) RT-PCR from RNA extracts of the *vamp721vamp722* mutants. The expression of *VAMP721* and *VAMP722* was not detected in the double mutants. Two biological replicates were performed. (C) 5-d-old wild-type and *vamp721vamp722* mutant seedlings derived from *vamp721^+/-^vamp722^-/-^* plants are shown. Bars  =  2 mm. (D) and (E) Root tip regions in 5-d-old wild-type (D) and *vamp721vamp722* mutant seedlings (E) stained with propidium iodide. Note the disorganized cell files in the mutant. Bars  =  20 µm. (F) 5-d-old wild-type and *vamp721vamp722* mutant seedlings under dark growth are shown. Bars  =  500 µm.

No obvious differences in plant growth were observed among single *vamp721*, *vamp722* mutants, heterozygous double mutants and wild-type control plants (Figure S1). Interestingly, we identified *vamp721vamp722* double homozygous mutant seedlings in the progeny of self-fertilized heterozygous double mutant plants based on genotyping and RT-PCR. (Figure 1A–1C and Figure S1). As shown in Figure 1C and 1F, the *vamp721vamp722* seedlings arrested 2 d after germination and grew extreme rudimentary roots, hypocotyls, and cotyledons, thus leading to seedling death 10 days later. Moreover, compared with wild-type seedlings, v*amp721vamp722* mutant roots exhibited disorganized root tips including abnormal meristematic cells and root caps (Figure 1D and 1E). The incorporation of *GFP-VAMP721* translational fusion gene under control of the native promoter into *vamp721^-/-^ vamp722^+/-^* plants fully rescued the seedling lethality phenotype in the double homozygous mutant, confirming that the phenotypic alterations were due to the T-DNA insertions in the two genes (Figure S1).

### *vamp721vamp722* mutant seedlings exhibit cell wall stubs and incomplete cytokinesis

**Figure 2:**
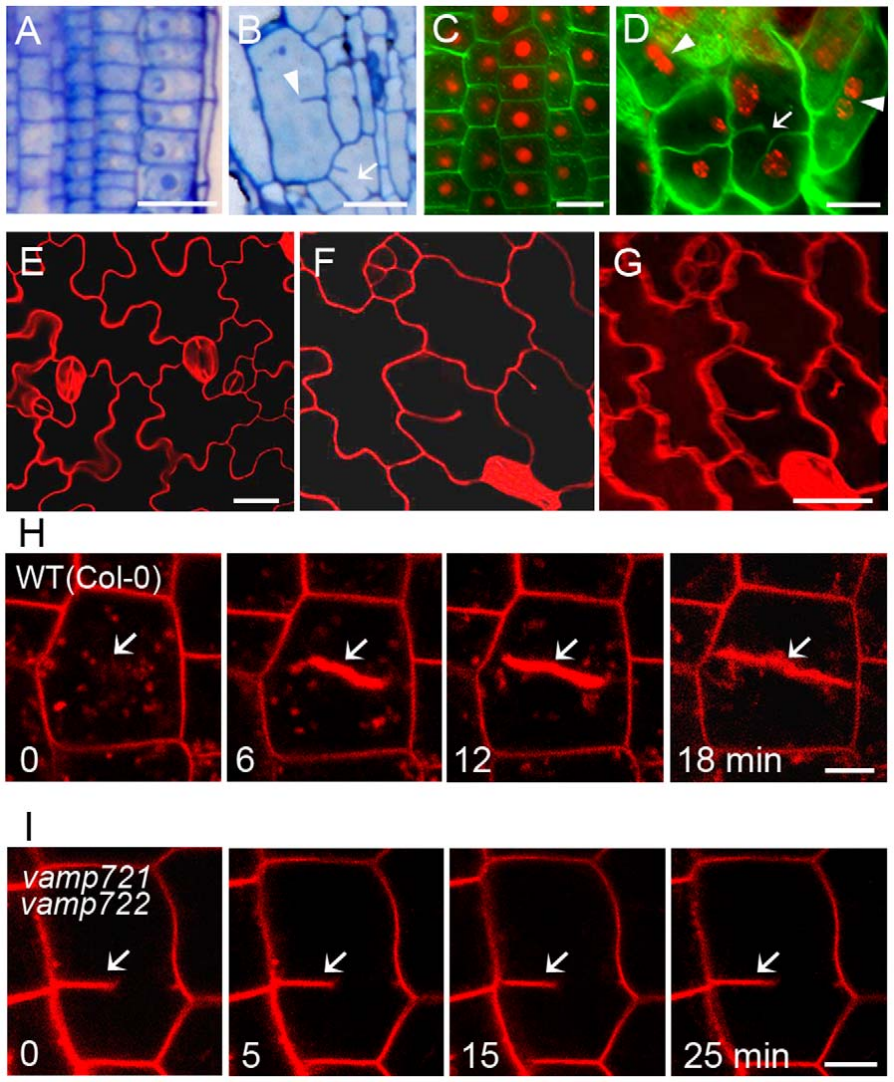
Cell wall stubs and incomplete cytokinesis in *vamp721vamp722* mutants. (A) and (B) Toluidine blue O-stained root longitudinal sections of wild-type (A) and *vamp721vamp722* seedlings (B). Cell wall stub (arrowhead) and gap (arrow) were observed in double mutants. Bars  =  20 µm. (C) and (D) Confocal images of propidium iodide-stained nuclei (red) and Calcofluor-stained cell walls (green) in root tip cells of 3-d-old seedlings. *vamp721vamp722* mutants showed incomplete cytokinesis (D) compared with that of wild type (C). Arrowheads indicate two nuclei in a single cell; arrow indicates discontinuous cell wall. Bars  =  10 µm. (E–G) Epidermal cells of cotyledons visualized using propidium iodide staining. Compared with wild type (E), cell wall stubs were observed in cotyledons of mutants (F), which was confirmed by 3D projection (G). Bars  =  20 µm. (H) and (I) Time-course analysis of growing cell plates stained with FM4-64 in root tips of 3- to 5-d-old wild-type (H) and *vamp721vamp722* (I) seedlings. Arrows indicate the growing or arrested cell plates. Bars  =  5 µm (H); 10 µm (I).

To gain insights into VAMP721 and VAMP722 function, the root longitudinal sections were prepared and compared. As a result, *vamp721vamp722* mutant seedlings exhibited disordered cell file alignment with cell wall stubs or gaps in comparison with wild-type plants (Figure 2A and 2B), implying that the cell division patterns were severely affected. When root tips were stained with Calcofluor and propidium iodide for cell walls and nuclei respectively, 97.8% of root cells in wild-type seedlings displayed normal cytokinesis characterized by one nucleus within a single cell and complete cell walls (Figure 2C and Table S2). However, the root cells of *vamp721vamp722* seedlings displayed a high incidence of binucleate cells and ruptured cell walls with the frequencies of 34.2% and 19.0%, as opposed to 1.3% and 0.9% in the wild-type plants (Figure 2D and Table S2). Similar to the wild type, 96.5% of root cells in complemented double mutant showed normal cytokinesis (Table S2). Furthermore, cell wall stubs were frequently observed in the cotyledon epidermal cells of *vamp721vamp722* mutants compared to wild type seedlings (Figure 2E–2G).

To determine whether loss function of VAMP721 and VAMP722 affected cell plate expansion, we traced the process of cell plate formation using time-lapse analysis by staining root tips of wild-type and *vamp721vamp722* mutant seedlings with FM4-64. As shown in Figure 2H, membrane structures in root cells stained with FM4-64 surrounded the cell-division plane prior to cell plate emergence in wild-type seedlings. After 6 min, a cell plate labeled with FM4-64 was apparently observed at the division plane. During the membrane fusion process, two edges of the cell plate continually expanded outward and finally anchored to parental membrane at 18 min. In contrast, root cells of *vamp721vamp722* seedlings exhibited asymmetric initiation of the cell plate at the beginning. Cell plate expansion was not detected after increasing the FM4-64 staining duration. Even after 25 min, cell plates in *vamp721vamp722* root cells slightly or rarely expanded (Figure 2I).

### VAMP721 and VAMP722 are localized to the cell plate during cytokinesis

**Figure 3:**
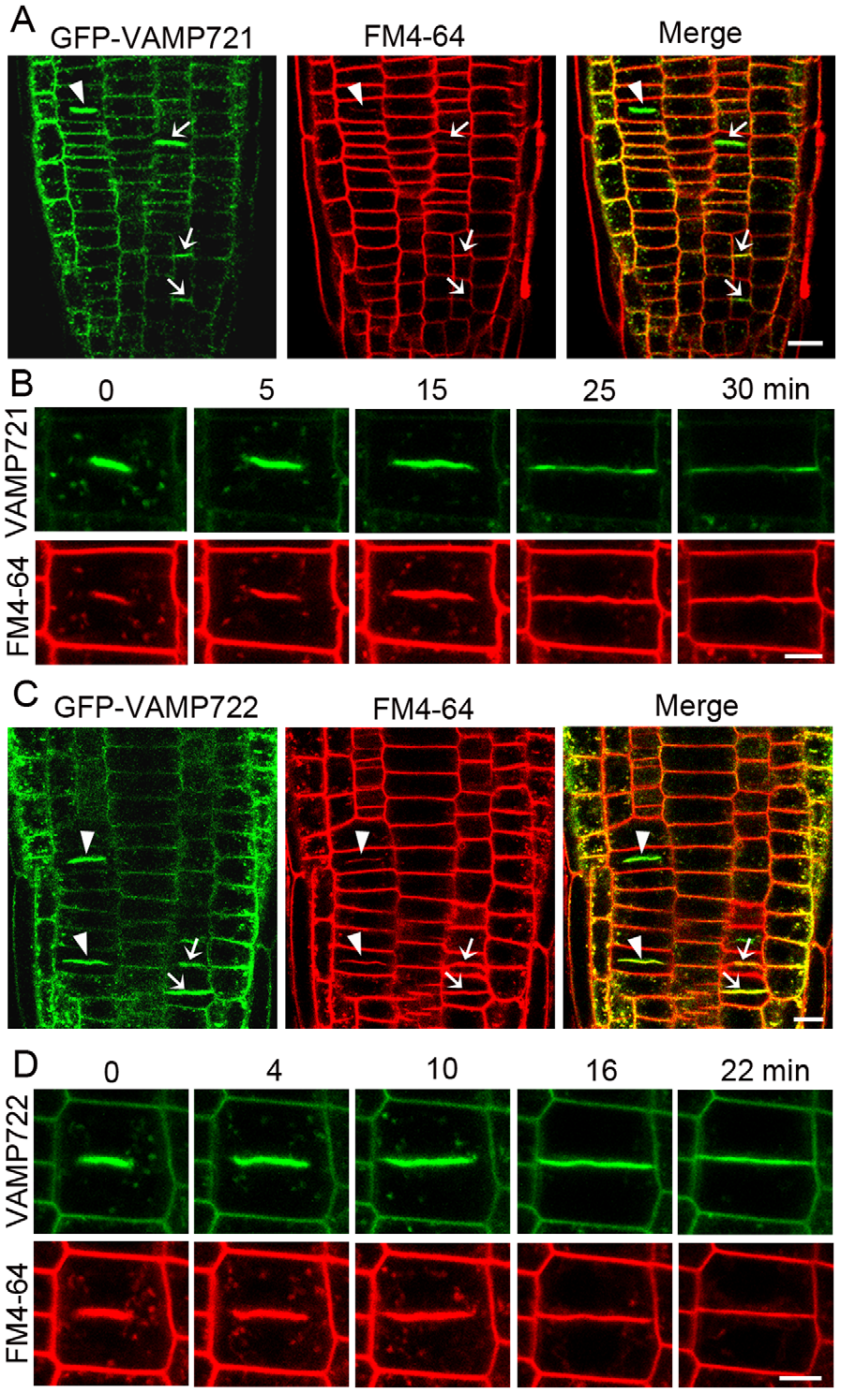
GFP-tagged VAMP721 and VAMP722 are localized to the cell plates and postcytokinetic walls. (A) and (C) GFP-VAMP721 (A) and GFP-VAMP722 (C) label the expanding cell plates (arrowheads) and postcytokinetic walls (arrows), which are merged with the membrane labeled with FM4-64. Bars  =  10 µm. (B) and (D) Time series of growing cell plates in root tip cells stained with FM4-64. GFP-VAMP721 (B) and GFP-VAMP722 (D) signals are localized to the cell plates during the progression of cytokinesis. Bars  =  5 µm.

To examine the subcellular localization of VAMP721 and VAMP722, we created transgenic plants in which N-terminal GFP or mCherry tagged *VAMP721* and *VAMP722* translational fusions were expressed respectively under the control of their native promoters in wild type (Col-0) background. Under the confocal microscope, we found that both GFP-VAMP721 and GFP-VAMP722 obviously appeared at expanding cell plates and postcytokinetic walls, which were merged with FM4-64-labeled membranes in root meristematic cells (Figure 3A and 3C). We also found a similar labeling pattern in cotyledon epidermal cells (Figure S2). To get deep insight into the GFP-VAMP721 and GFP-VAMP722 cytokinetic localization, the root meristematic cells of 3- to 5-d old *Arabidopsis* seedlings were monitored to visualize the process of cell plate formation. During the onset of cytokinesis, the GFP-VAMP721 signal was strongly associated with the newly formed cell plate at the middle of the division plane, and the organelles labeled with GFP-VAMP721 surrounded the cell plate (Figure 3B). Along with the process of cytokinesis, GFP-VAMP721 substantially accumulated at the entire cell plate and two edges of the cell plate expanded symmetrically outward before anchoring with the mother wall. After complete fusion of both cell plate edges with the parental PM, GFP-VAMP721 still remained associated with the postcytokinetic wall, and its intensity gradually decreased to the level of the surrounding cells after 30 min (Figure 3B).

As expected, GFP-tagged VAMP722 displayed a similar cytokinetic localization pattern to GFP-VAMP721. As shown in Figure 3D, the GFP-VAMP722 signal was apparently associated with the emerging cell plate at the division plane soon after the onset of cytokinesis. Membrane organelles labeled with GFP-VAMP722 were distributed in the vicinity of the cell plate during the process of cytokinesis. Two edges of the cell plate attached simultaneously to the parental PM at 16 min. Soon after that, the cell plate completed fusion with the plasma membrane, and the GFP signal decreased and appeared evenly along the entire cell plate (Figure 3D).

### The complemented double mutant reestablishes the proper cytokinesis

**Figure 4:**
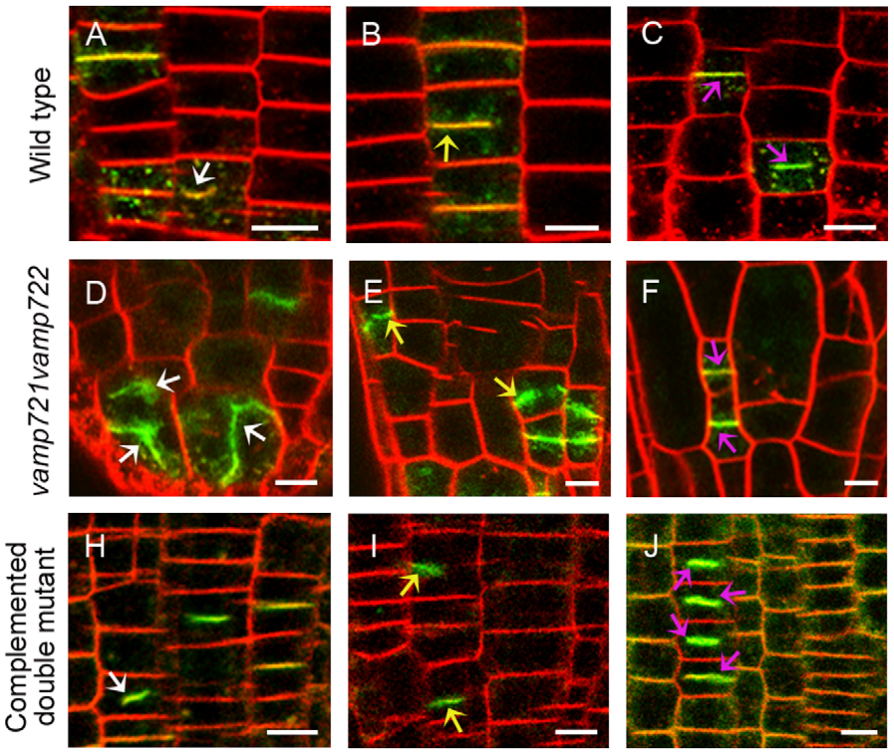
Cell plate formation during cytokinesis in control, *vamp721vamp722* and complemented double mutant root tips. Confocal analysis of cell plate formation monitored by GFP signals (green) together with FM4-64 staining (red) in cytokinetic root tip cells. White arrows indicate abnormal cell plates with irregular direction and/or thickness; yellow arrows indicate asymmetric expansion of cell plate and pink arrows indicate symmetric expansion or complete cell plate formation. (A–C) The cell plate formation in GFP-KNOLLE seedlings used as the control. Bars  =  10 µm. (D–F) The cell plate formation in *vamp721vamp722* seedlings labeled with cell plate marker GFP-KNOLLE. Bars  =  10 µm. (H–J) The cell plate formation in complemented double mutant seedlings rescued by *pVAMP721::GFP-VAMP721*. Bars  =  10 µm.

We further quantified the phenotypes of cell plate formation in cytokinetic root tip cells of control, *vamp721vamp722* and complemented double mutant seedlings by the fluorescent labeling (Figure 4 and Table S3). As expected, colocalization at the cell plates and postcytokinetic walls was observed in the root cytokinetic cells co-expressing cytokinesis-specific marker GFP-KNOLLE and mCherry-VAMP721 (Figure S3). Therefore, we used the GFP-KNOLLE transgenic lines as the control. The results showed that 96.9% of cell plates in control cells exhibited symmetric assembly or complete formation. However, abnormal and asymmetric cell plate assemblies were scored highly in 41.8% and 10.9% of *vamp721vamp722* cytokinetic root cells labeled with GFP-KNOLLE, consistent with the observation that the double mutant frequently showed disordered cell file alignment and cell wall stubs. Similar to the control, 94.3% of the mitotic root cells in complemented double mutant showed normal cell plate formation monitored by GFP-VAMP721.

### VAMP721 and VAMP722 define the trans-Golgi/early endosomal compartments

**Figure 5:**
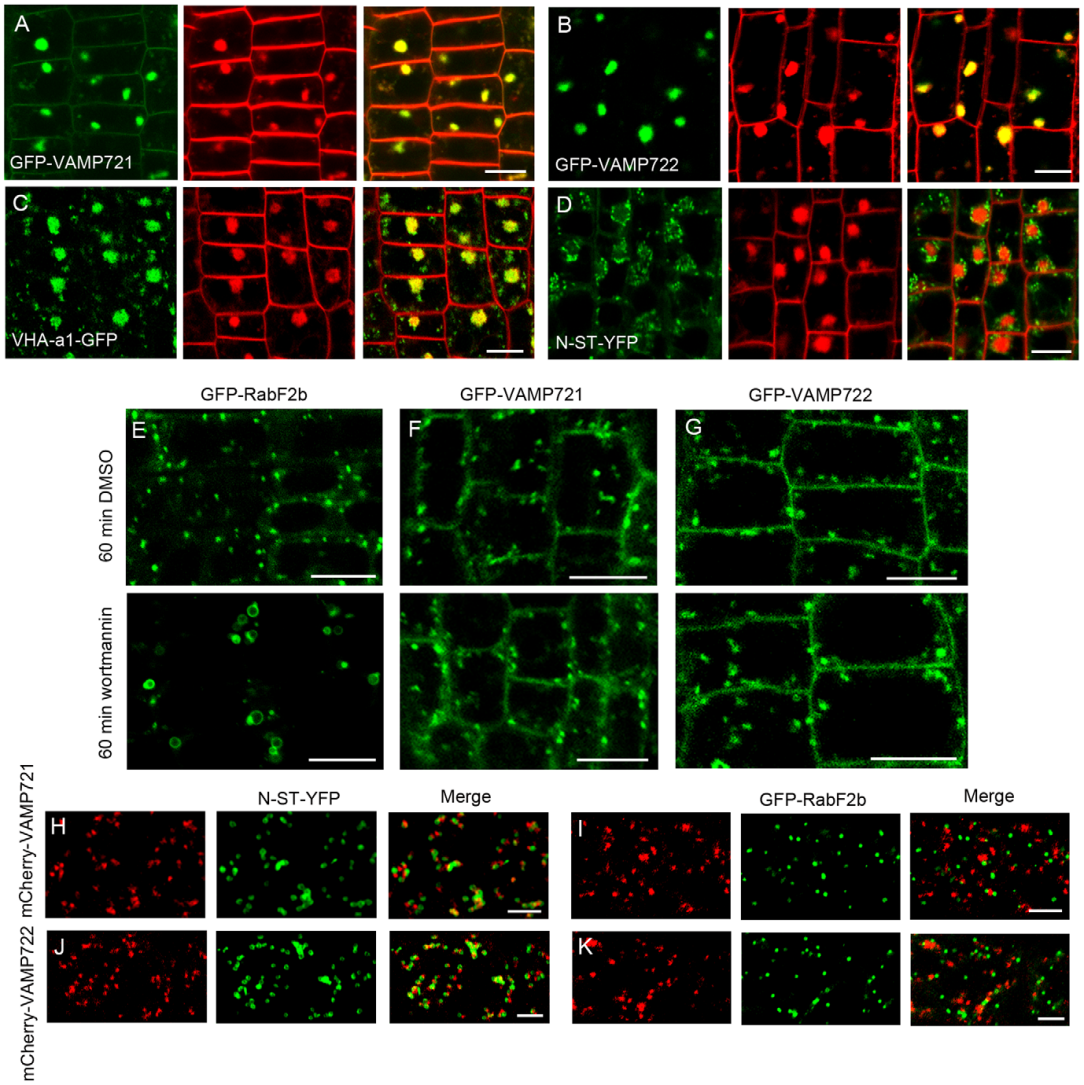
VAMP721- and VAMP722-labled organelles are distinct from the Golgi and PVC. (A–D) 5-d-old seedlings were pre-incubated in 50 µM BFA for 30 min before incubation in 50 µM BFA plus 5 µM FM4-64 for another 30 min. (A) and (B) GFP-VAMP721 (A) and GFP-VAMP722 (B) relocated to BFA compartments labeled with FM4-64. Bars  =  10 µm. (C) VHA-a1-GFP-labeled TGN was sensitive to BFA treatment and colocalized with BFA compartment from FM4-64. Bars  =  10 µm. (D) FM4-64 was accumulated into the core of BFA compartment surrounded by the Golgi marker N-ST-YFP after BFA treatment. Bars  =  10 µm. (E–G) Seedlings were incubated with 33 µM wortmannin for 60 min. DMSO was used as the control. Bars  =  10 µm. (E) In contrast to the control, the PVC marker GFP-RabF2b was induced to form small vacuoles after wortmannin treatment. Bars  =  10 µm. (F) and (G) Similar to the DMSO control, the organelles labeled with (F) GFP-VAMP721 and (G) GFP-VAMP722 were not changed following wortmannin treatment. Bars  =  10 µm. (H–K) Confocal laser scanning microscopy (CLSM) analysis of seedling root epidermal cells co-expressing the Golgi marker N-ST-YFP or PVC marker GFP-RabF2b as indicated (green) and mCherry-VAMP721 or mCherry-VAMP722 as indicated (red). Co-localization analysis showed that mCherry-VAMP721- and mCherry-VAMP722-labeled organelles were closely associated with Golgi stacks in (H) and (J). Similarly, as shown in (I) and (K), mCherry-VAMP721- and mCherry-VAMP722-labeled organelles were also distinct from the PVC marker GFP-RabF2b. Bars  =  5 µm.

Apart from the cell plate localization, we also found that GFP-VAMP721 and GFP-VAMP722 were frequently distributed to the cytoplasmic endosomes (Figure S4). To define the endosomes labeled with GFP-VAMP721 and GFP-VAMP722, we first use the trafficking inhibitor Brefeldin A (BFA), a fungal macrocyclic lactone, which targets (guanine nucleotide exchange factors for ARF GTPases) ARF-GEFs, thus inhibiting the function of ARF GTPases and induces heterogeneous aggregations (so called BFA compartments) of early endosomal membranes in plants [19], [20], [21]. When root tips were treated with 50 µM BFA plus FM4-64, the endosomes labeled with GFP-VAMP721 were induced to form aggregates that colocalized with positive BFA compartments labeled with endocytic FM4-64 (Figure 5A). Similar results were obtained when GFP-VAMP722-labeled root tip cells were treated with BFA (Figure 5B). We further found that BFA treatment induced membrane aggregates labeled with VHA-a1-GFP, a TGN/early endosome marker (Figure 5C). However, the Golgi marker N-ST-YFP did not enter the BFA compartments, but instead surrounded FM4-64-enriched BFA compartments (Figure 5D).

To determine whether the endosomes labeled with GFP-VAMP721 and GFP-VAMP722 belong to the prevacuolar compartment (PVC)/late endosome (LE), we further used wortmannin, an inhibitor of phosphatidylinositol-3 kinase, which targets the PVC/LE that then dilates and blocks the traffic to vacuole in plants [22], [23]. After treatment with wortmannin at 33 µm for 60 min in the transgenic root tip cells, we observed that the PVC marker GFP-RabF2b was induced to form small vacuoles, which are the representative wortmannin treatment structures (Figure 5E). In contrast, wortmannin treatment did not cause visible changes of the organelles labeled with GFP-VAMP721 and GFP-VAMP722 in size or number, similar to the results in DMSO control (Figure 5F and 5G). In the root cells co-expressing mCherry-tagged VAMP721 and fluorescence marker of Golgi, mCherry-VAMP721-labeled organelles were in physical proximity with the Golgi marked with N-ST-YFP (Figure 5H). For root cells co-labeled with mCherry-VAMP721 and the PVC marker GFP-RabF2b, we found that the organelles labeled with mCherry-VAMP721 were often transiently close to, but distinct from the PVC (Figure 5I). Similarly, the organelles labeled with mCherry-VAMP722 were distinct from the Golgi apparatus and PVC markers (Figure 5J and 5K).

**Figure 6:**
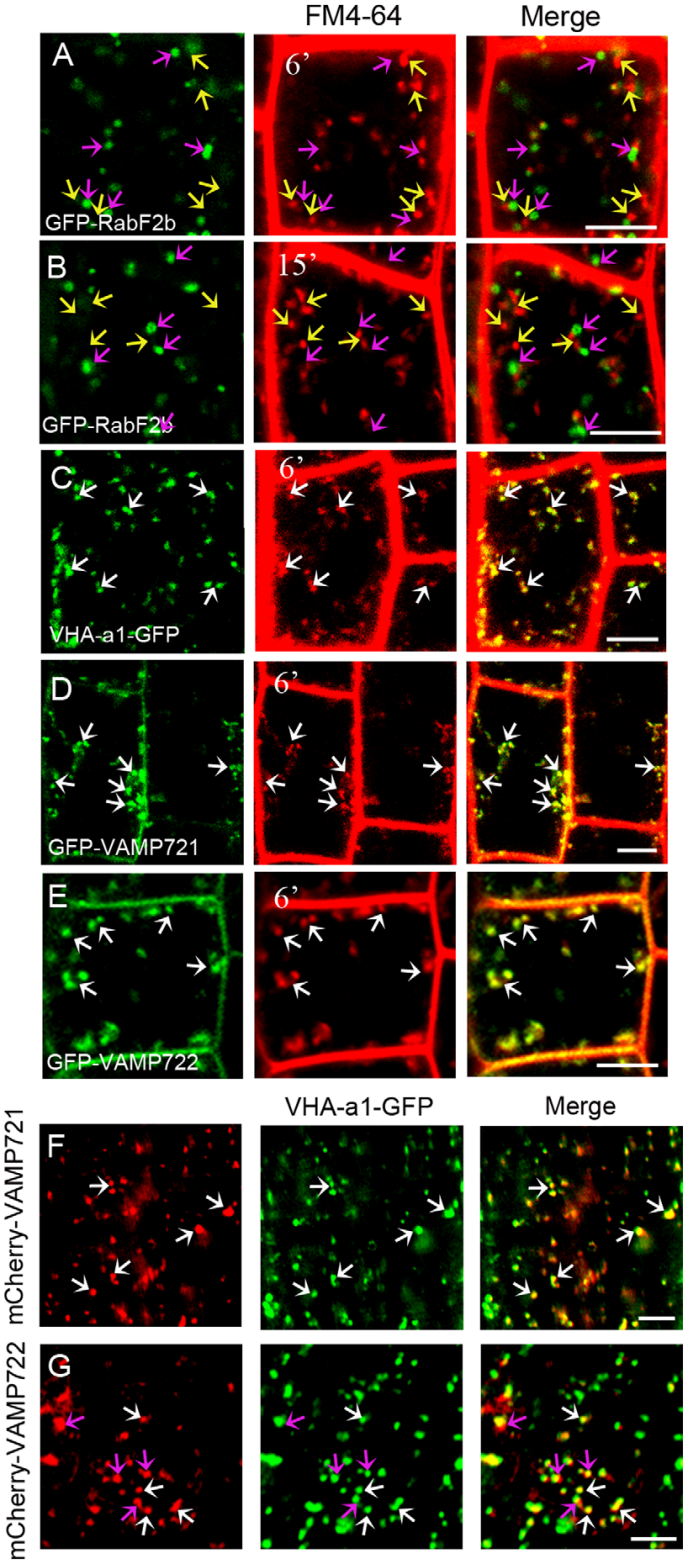
VAMP721- and VAMP722-labeled organelles are rapidly labeled by internalized FM4-64 and overlap the VHA-a1 TGN. (A–E) Root tip cells expressing VHA-a1-GFP (A), GFP-RabF2b (B) and (C), GFP-VAMP721 (D), or GFP-VAMP722 (E) (each green) incubated with FM4-64 (red) for the times indicated. Pink arrows indicate GFP-labeled regions without detectable FM4-64 labeling; yellow arrows indicate FM4-64–labeled regions without detectable GFP signal; white arrows indicate regions with extensive colocalization between GFP and FM4-64 signals. Bars  =  5 µm. (F) and (G) Confocal laser scanning microscopy analysis of root epidermal cells coexpressing VHA-a1-GFP (green) and (F) mCherry-VAMP721 (red) or (G) mCherry-VAMP722 (red). Bars  =  5 µm.

In *Arabidopsis*, lipophilic styryl dye FM4-64 is internalized from plasma membrane to lytic vacuole within 1 to 2 h via passing through a variety of endosomes along the endocytosis [24], [25], [26]. When FM4-64 was applied to root tips expressing the early endosome marker VHA-a1-GFP, extensive colocalization was observed in epidermal cells after uptake for 6 min (Figure 6A). In contrast, the internalized FM4-64 did not colocalize with PVC labeled with GFP-RabF2b after 6 min, although they were adjacent to each other (Figure 6B). Even after 15 min, GFP-RabF2b-labeled PVC showed very limited colocalization with the internalized FM4-64 (Figure 6C). However, internalized FM4-64 colocalized mostly with transgenes-labeled endosomes after 6 min in cells expressing GFP-VAMP721 or GFP-VAMP722, similar to the labeling pattern of the VHA-a1-GFP compartment (Figure 6D and 6E). To unveil the spatial relationship between the VAMP721/VAMP722 and VHA-a1 compartments, we crossed plants expressing mCherry-VAMP721 or mCherry-VAMP722 with the VHA-a1-GFP lines. Under the confocal microscope, we observed that mCherry-VAMP721 and VHA-a1-GFP exhibited overlapping membrane distributions (Figure 6F). Similarly, fluorescence signals from mCherry-VAMP722 were colocalized with those of VHA-a1-GFP (Figure 6G).

### Inhibiting traffic at the trans-Golgi network affects cell plate formation

**Figure 7:**
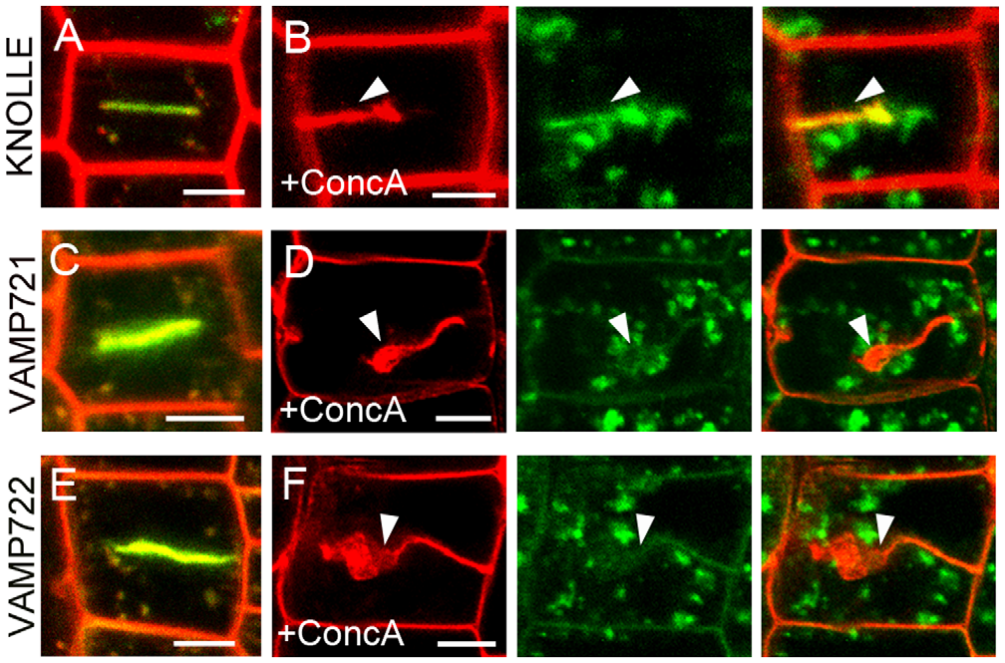
Concanamycin A interferes with cell plate formation. (A–F) Fluorescence imaging of root cells. The cell outlines were stained with FM4-64. Compared with the expanding cell plates in controls (A), (C) and (E), ConcA treatment for 2 h induced intracellular accumulation of GFP-KNOLLE (B), GFP-VAMP721 (D), and GFP-VAMP722 (F), and prevented the cell plate formation indicated with arrowheads in (B), (D), and (F). Bars  =  5 µm.

ConcanamycinA (ConcA) is a membrane-permeable macrolide antibiotics that binds to the V-ATPase subunits c and inhibits proton transport [27]. It has been demonstrated that ConcA blocks trafficking at the TGN, which affects cell plate formation in *Arabidopsis* [21], [28], [29]. When ConcA was applied to root tip cells, the organelles labeled with GFP-KNOLLE were clearly trapped in cytosolic aggregates (Figure 7B and Figure S5). As expected, the ConcA treatment retarded the expansion of the cell plate labeled with GFP-KNOLLE, leading to cell wall stub, as shown in Figure 7B. Similarly, we found that ConcA treatment induced a massive intracellular accumulation of the endosomes labeled with GFP-VAMP721 and GFP-VAMP722 (Figure 7D, 7F and Figure S5). Furthermore, cell wall stubs with irregular direction and thickness surrounded with swollen structures were observed in ConcA-treated root dividing cells expressing GFP-VAMP721 and GFP-VAMP722 (Figure 7D and 7F). However, these fluorescent signals were localized to the normal expanding cell plates in untreated dividing cells (Figure 7A, 7C and 7E).

### VAMP721 and VAMP722 are required for secretory trafficking to the plasma membrane

**Figure 8:**
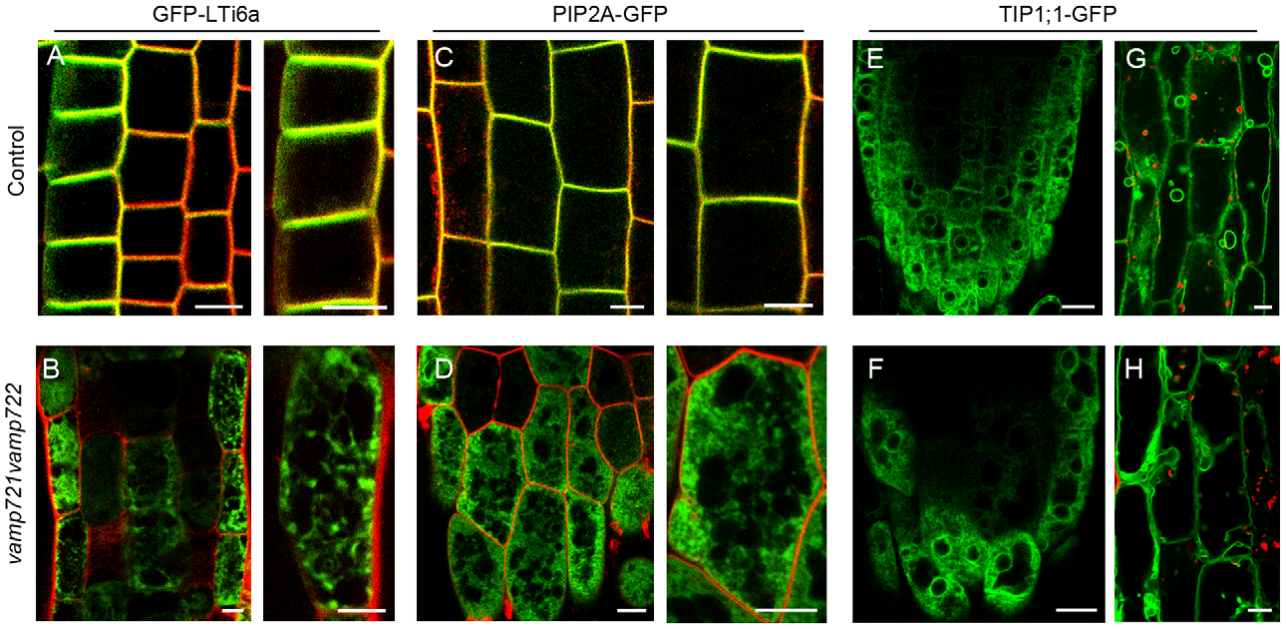
Localization of the plasma membrane and tonoplast markers in control and *vamp721vamp722* mutant seedlings. (A) GFP-Lti6a localization in control root. Note (enlargement at right) that GFP-Lti6a remained extensively colocalized with FM4-64 at the plasma membrane. Bars  =  10 µm. (B) GFP-Lti6a localization in *vamp721vamp722* roots. Note (enlargement at right) that GFP-Lti6a abnormally accumulated in the cytoplasm. Bars  =  10 µm. (C) PIP2A-GFP localization in control roots. Note (enlargement at right) that PIP2A-GFP remained extensively colocalized with FM4-64 at the plasma membrane. Bars  =  10 µm. (D) PIP2A-GFP localization in *vamp721vamp722* roots. Note (enlargement at right) that PIP2A-GFP accumulated inside aberrant intracellular compartments. Bars  =  10 µm. (E) and (F) TIP1;1-GFP showed similar tonoplast labeling patterns in cells of control (E) and *vamp721vamp722* (F) root tips. Bars  =  10 µm. (G) and (H) TIP1;1-GFP-labeled tonoplast and small vacuoles in cells of control (G) and *vamp721vamp722* (H) hypocotyls. Red signals represent chloroplast autofluorescence. Bars  =  10 µm.

The role of *de novo* secretory trafficking in plant cytokinesis has been emphasized [28]. The occurrence of incomplete cell plate in *vamp721vamp722* seedlings and their plasma membrane localization imply that VAMP721 and VAMP722 probably are involved in the secretory trafficking to the plasma membrae. In *vamp721vamp722* mutant seedlings expressing plasma membrane marker protein GFP-Lti6a, we observed an abnormal accumulation of GFP signals in the cytoplasm of root epidermal cells (Figure 8B). Even at a higher resolution, we did not detect colocalization between GFP-Lti6a and FM4-64 staining at the plasma membrane (Figure 8B). Similarly, we observed GFP signals inside aberrant intracellular compartments in *vamp721vamp722* roots expressing another PM marker, PIP2A-GFP (Figure 8D). In contrast, GFP-Lti6a and PIP2A-GFP showed clear PM localization overlapping with the FM4-64 staining at low or high magnification in control plants (Figure 8A and 8C). However, the cells expressing TIP1;1-GFP in *vamp721vamp722* mutant displayed similar tonoplast labeling patterns to that of controls in roots and hypocotyls (Figure 8E–8H).

## Discussion

SNARE molecules play important roles in cell-plate vesicle fusion during plant cytokinesis [12]. Based on the sequence information from available genome assemblies, *VAMP721* and *VAMP722* are classified into R-SNAREs of the VAMP72 group [13]. Recent studies implicate that VAMP721 and VAMP722 are essential for plant growth in other aspects in addition to their roles in plant immune responses [30]. In the present study, our results suggest that VAMP721 and VAMP722 are essential R-SNARE molecules required for cell plate formation. This conclusion is based on four key findings: (1) the homozygous *vamp721vamp722* double mutant exhibited a strong cytokinesis-defective phenotype, lethal dwarf seedlings, characterized by the frequent appearance of bi-nucleate cells, cell wall stubs, or gaps. However, we did not detect any seedling-level cytokinetic defects in single *vamp721* or *vamp722* mutants or in heterozygous double mutants (Figure S6), indicating that VAMP721 and VAMP722 have redundant functions in cytokinesis; (2) *vamp721vamp722* mutations retarded cell plate expansion; (3) Confocal images revealed that both GFP-tagged VAMP721 and VAMP722 were localized to both the growing cell plates with strong signals and postcytokinetic walls with decreased intensity during cytokinesis. These labeling patterns are different from those of exocyst components that started to substantially accumulate at cell plate insertion sites after fusion with the mother wall [31]; and (4) The resumed plant growth of complemented double mutant was probably due to the reestablishment of proper cytokinesis. Yet, we can not explain the precise mechanism leading to the incomplete cell wall phenotype in the *vamp721vamp722* mutant. The most probable explanation is that VAMP721 and VAMP722 mediate homotypic membrane fusion during the entire process of cytokinesis. In this scenario, vesicle fusion is severely impaired when the key components of R-SNARE are absent [32]. Alternatively, VAMP721 and VAMP722 may mediate heterotypic fusion of later-arriving vesicles with the nascent cell plate, which attributes to a VAMP721- and VAMP722-independent mechanism. However, such a two-step formation of cell plate during cytokinesis has not been documented. In any case, the fact that VAMP721 and VAMP722 contribute to the cell plate maturation is beyond doubt. These results suggest that VAMP721 and VAMP722 are the newly identified R-SNARE components for cell-plate membrane fusion.

Charting the subcellular localization facilitates the functional analysis of the interested genes. However, few studies deal with the subcellular localization and trafficking of VAMP721 and VAMP722 in detail except the early data that these two proteins are localized to plasma membrane and unknown organelles in *Arabidopsis* protoplasts by transient assays [33]. In our present study, we confirmed the PM localization of VAMP721 and VAMP722 in *Arabidopsis* root cells. Importantly, we demonstrated that both VAMP721 and VAMP722 were localized to the cell plate during cytokinesis and cytosolic TGN/early endosomal compartments overlapped with VHA-a1-labled TGN domains. BFA treatment induces the accumulation of endocytosed proteins, FM4-64 and early endosome markers such as VHA-a1 and RabA2/A3 in large aggregates of membranes referred to as BFA compartments in *Arabidopsis* [21], [25], [34]. Our results showed that when FM4-64 was added to root cells pretreated with BFA, FM4-64 accumulated into the BFA compartments in a region enriched with VHA-a1 proteins and surrounded by the trans-Golgi marker N-ST-YFP. Furthermore, we found that endosomes labeled with GFP-VAMP721 or GFP-VAMP722 were induced to accumulate at the core of BFA compartments that were enriched with FM4-64 after BFA treatment, indicating that GFP-VAMP721 and GFP-VAMP722-labeled endosomes are sensitive to BFA treatment, similar to the response of the VHA-a1 compartment.

The late enodosome markers were very sensitive to wortmannin treatment and the inhibitory effect of wortmannin can be monitored by the Rab GTPase RabF2b, as it is the most accepted PVC marker [28], [35], [36]. In contrast to the response of GFP-RabF2b, wortmannin treatment did not induce vacuolation of endosomes labeled with GFP-VAMP721 and GFP-VAMP722, indicating that GFP-VAMP721- and GFP-VAMP722-labeled endosomes were not the targets of wortmannin. The co-labeling experiments using plants expressing mCherry-tagged VAMP721 or VAMP722 and the trans-Golgi marker N-ST-YFP or the PVC marker GFP-RabF2b showed that both the VAMP721 and VAMP722 compartments were distinct from the marker-labeled Golgi stacks and the PVC, although they were often close to these organelles. Deducing from the effects of the trafficking inhibitors on transgenes-labeled endosomes and the colocalization analysis, we can conclude that the organelles labeled with GFP-VAMP721 and GFP-VAMP722 do not behave as typical Golgi apparatus and PVC, providing clues for early endosomal compartments.

FM4-64 has been demonstrated as a useful tool to chart the endocytic pathway in plant cells [37], [38], [39], [40]. Our double labeling experiments showed that FM4-64 rapidly and significantly accumulated in the VAMP721 and VAMP722 compartments before it reached the PVC labeled with GFP-RabF2b within 6 min, similar to the labeling pattern of the VHA-a1-GFP compartment. Our results are consistent with recent findings that VHA-a1 and Rab-A2/A3 compartments are the early sites of FM4-64 labeling within 5–6 min, whereas the PVC compartment identified by BP80 or the Rab-F2 subclass apparently separates from the FM4-64 signal within the same time and even after 10 min or more after dye application in *Arabidopsis* root tip cells [21], [25]. These results suggest that the VAMP721 and VAMP722 compartments also represent early sites of FM4-64 accumulation. The colocalization analyses showed that the VAMP721 and VAMP722 compartments colocalized with VHA-a1-labeled TGN membrane domain, indicating that VAMP721 and VAMP722 define the TGN/early endosomal compartments that either give rise to or are derived from compartments that carry VHA-a1 proteins. Thus, the TGN/early endosomal compartment localization of VAMP721 and VAMP722 might imply the putative trafficking pathways mediated by these two proteins either trafficking from Golgi to TGN or internalization from PM to TGN.

Our results also confirmed the inhibitory effect of ConcA on post-TGN trafficking and cell plate formation in dividing cells [28], [29]. Using this inhibitor, we found that GFP-KNOLLE-labeled vesicles were induced to form aggregates, resulting in an incomplete cell wall, similar to previous results [28]. We also observed that ConcA treatment induced cellular accumulation of GFP-VAMP721- and GFP-VAMP722-labeled organelles and impaired the maturation of cell plate in dividing cells. Our results indicate that the vesicles and endosomes labeled with GFP-VAMP721 and GFP-VAMP722 are indeed transported from the TGN to the division plane during cytokinesis, similar to the trafficking pathway of the cell plate marker GFP-KNOLLE. Moreover, the evidence presented here highlights the specialized TGN function in mitotic cells, which has been strongly suggested by other studies in *Arabidopsis*. For example, KNOLLE positive vesicles move to the cell plate through the TGN during cytokinesis. After cell plate formation, KNOLLE is retrieved to PVC, possibly via TGN, and finally to the lytic vacuole for degradation [28]. Additionally, Chow et al. [25] revealed that the small GTPases RAB-A2/A3 proteins, which define a new TGN/early endosomal membrane domain, colocalized with KNOLLE throughout mitosis and contributed substantially to the cell plate. Recently, it was shown that the MPK6 localized to the secretory TGN vesicles is involved in cell division plane control [41]. Thus, our findings suggest that the VAMP721- and VAMP722-labeled vesicles and endosomal compartments sorted from TGN/early endosomal membrane domains are required for cell plate construction.

**Figure 9:**
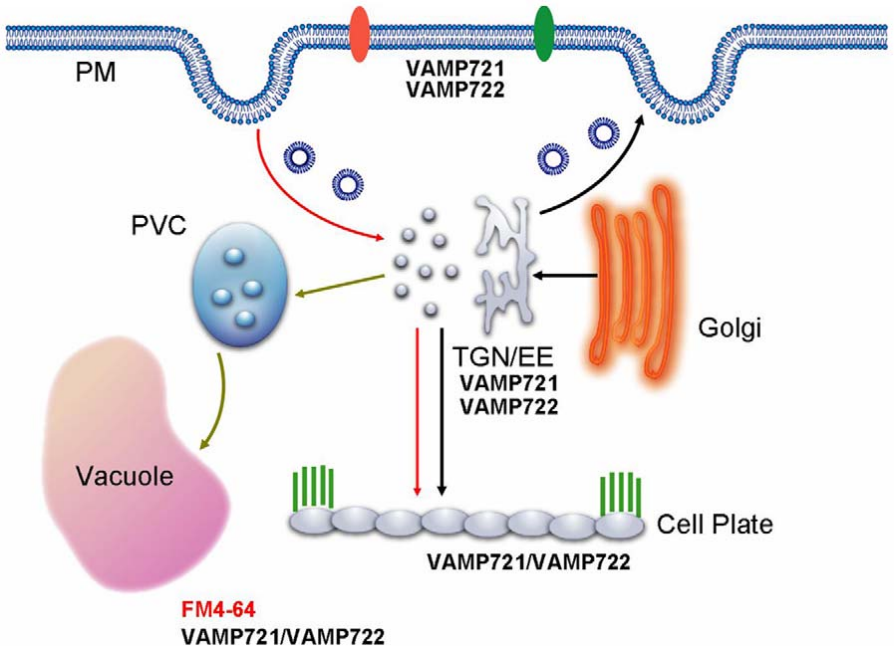
Hypothetical model of vesicle trafficking during cell plate formation in root tip cells of *Arabidopsis*. Based on this study and previous results, secretory (black arrow) and endocytic (red arrow, such as FM4-64) trafficking pathways converge at the TGN/EE to reach the cell plate. VAMP721 and VAMP722 compartments mediate the traffic of Golgi-derived vesicles to cell plate via TGN/EE membrane domain. In addition, VAMP721 and VAMP722 compartments mediate protein secretion to the plasma membrane. PM, plasma membrane; TGN, trans-Golgi network; EE, early endosome; PVC, prevacuolar compartment.

It has long been accepted that the newly synthesized material from Golgi apparatus-originated secretory vesicles mainly contributes to the cell plate formation. Inhibition of ER-Golgi trafficking with BFA treatment suppressed the transport of newly synthesized KNOLLE from Golgi to the cell plate via TGN and resulted in binucleate cells and cell wall stubs in *gnl1* seedlings [28]. RAB-A2/A3 compartment lay on the secretory pathway from Golgi to plasma membrane and dominant-inhibitory mutants of RAB-A2^a^ prolonged the retention at Golgi or plasma membrane, thus impairing cytokinesis by titrating their interactors [25]. Golgi-derived membrane and proteins, however, are not the only source for cell plate construction. In BY-2 cells and *Arabidopsis* seedlings, the endocytic tracers FM4-64 or the fluid phase markers Alexa 633 and Lucifer Yellow clearly labeled the forming cell plate within minutes after addition [21], [42]. Moreover, several PM marker proteins and parental cell wall-derived pectins were found to internalize and target into cytokinetic cell plate, in parallel with an increasing rate of endocytosis when the cell plate was forming [42], [43], supporting the role of the endocytic pathway in cell plate building. However, the relative contribution between secretory and endocytic trafficking to cell plate formation remained to be further determined. Our results showed that in *vamp721vamp722* mutant seedlings, the PM marker proteins were abnormally aggregated in the cytoplasm almost without plasma membrane localization, while the tonoplast marker proteins appeared normal localization, demonstrating that VAMP721 and VAMP722 are required for PM proteins trafficking and vesicle fusion at the plasma membrane. We also found that *vamp721vamp722* mutations retarded cell plate expansion, probably due to the impaired membrane fusion at the division plane. Given the link between secretion of PM proteins and membrane targeting during cytokinesis, our findings suggest that VAMP721 and VAMP722 are essential for vesicle delivery, in particular for vesicle fusion, at the cell-division plane to complete cell plate expansion during plant cytokinesis. Based on our results together with recent publications, a hypothetical model for vesicle trafficking during plant cytokinesis, in which VAMP721- and VAMP722-labeled TGN/early endosomal compartments converge the secretory and endocytic pathways, is presented in Figure 9.

## Materials and Methods

### Plant materials and growth conditions

All plants used for experiments were *Arabidopsis* Col-0.The homozygous *vamp721* (At1g04750), *vamp722* (At2g33120) mutants, SALK_037273, SALK_119149 and *vamp721^+/-^vamp722^-/-^*, *vamp721^-/-^ vamp722^+/-^* heterozygous double mutants were kindly provided by Paul Schulze-Lefert [30]. *vamp721vamp722* double mutants were isolated from progeny of heterozygous double mutants. Primers used for mutants genotyping are listed in Table S1. Plants expressing N-ST-YFP, VHA-a1-GFP, GFP-RABF2b, GFP-KNOLLE and GFP-LTi6a have been described previously [20], [21], [28], [36], [44]. For *vamp721vamp722* mutant seedlings expressing GFP-LTi6a, GFP-KNOLLE, F2 lines derived from crosses between GFP-LTi6a or GFP-KNOLLE lines and *vamp721^+/-^vamp722^-/-^* plants were genotyped to isolate the heterozygous double mutants containing the marker protein. For *vamp721vamp722* mutant seedlings expressing PIP2A-GFP and TIP1;1-GFP, the plasmids *PIP2A-GFP*(CD3-1003) and *TIP1;1-GFP* (CD3-971) [45] were used to transform the heterozygous double mutants. Wild-type looking seedlings from the mother plants were used as the controls.

For growing seedlings on agar-containing plates, *Arabidopsis* seeds were pretreated in 70% ethanol for 1 min, surface-sterilized in 2.5% bleach for 10 min, and washed with distillated water at least five times. Seeds were planted on 1% agar-containing 0.5x Murashige and Skoog Salts, 1% sucrose, pH 5.8, stratified and placed at 4°C in the dark for 2 d before germination. Growth conditions were at 23°C with a 16-h-light/8-h-dark cycle, either in soil or on MS plates.

### Constructs and plant transformation

For fluorescent fusion protein constructions, 1.8kb *VAMP721* promoter and 2.0kb *VAMP722* promoter before the start codon of each gene were amplified from genomic DNA of wild-type *Arabidopsis thaliana* ecotype Columbia plants and cloned into the pCAMBIA1300 binary expression vector with *Hin*dIII and *Sal*I respectively. To create the translational fusions of *VAMP721* or *VAMP722* tagged with GFP or mCherry, a cloning vector *pUC18/pCAMBIA1300-GFP-AtFim1ABD2* [46] was used. *GFP* sequence was replaced with cDNA encoding mCherry. The genomic sequences of *VAMP721* and *VAMP722* were PCR amplified and subcloned into the cloning vectors with *Spe*I and *Not*I replacing the *ABD2* fragment. Then the resulted cloning vectors were digested with *Sal*I and *Eco*RI and the *Sal*I-*Eco*RI fragments including *GFP/mCherry-VAMP721/VAMP722* were cloned into the pCAMBIA1300 under *VAMP721* or *VAMP722* promoter. Primer sequences used for the constructs are listed in Table S1. All The sequences cloned above were checked by sequencing. The binary vectors were transformed into *A. tumefaciens* strain GV3101. Transformation of *Arabidopsis* plants was performed by floral dipping using *Agrobacterium tumefaciens* [47]. The selection of transgenic lines was performed on 1/2 MS solid medium containing 3% sucrose with 25ug/ml hygromycin. For complementation the resulting *A.tumefaciens* transformant *pVAMP721::GFP-VAMP721* was used to transform *vamp721^-/-^ vamp722^+/-^* plants. The homozygous identity of T-DNA insertion of the rescued plants was confirmed by PCR assay in the T2 plants.

### RT-PCR analysis

Total RNA was extracted from seedlings of four-day-old wild-type and *vamp721vamp722* double mutant using SV Total RNA Isolation Kit (promega), and cDNA was synthesized with SuperScript III reverse transcriptase (Invitrogen). PCR conditions were as follows: 95°C (4 min); 28 cycles of 95°C (30 s), 57°C (30 s), 72°C (1 min), and 72°C (10 min). Primers used for RT-PCT are listed in Table S1.

### Cross-section analysis of wild-type and *vamp721vamp722* mutant roots

The root tissue from 4-day-old seedling was cut and immediately vacuum infiltrated and fixed with 2.5% (v/v) glutaraldehyde and 2% (v/v) paraformaldehyde in 0.1 M phosphate-buffered saline (pH 7.2) at room temperature for 4 h, followed by postfixation in 1%OsO_4_ buffer at 4°C overnight. Samples were subsequently rinsed with 0.1 M phosphate buffer and dehydrated through a graded ethanol series (30%–100%). Then, samples were embedded in LR White (EMS) and polymerized at 60°C for 24 h. Semithin (1 µm) sections were cut using an Ultracut microtome (EM UC6; Leica). Semthin sections were stained with toluidine blue O before observation.

### Fluorescent dye and inhibitor treatments

For FM4-64 staining, seedlings were incubated in half-strength MS liquid containing 5 µM FM4-64 (Invitrogen, diluted from a 5 mM stock in water) for a specified time at room temperature. To stain cell walls and nuclei simultaneously, Calcofluor and propidium iodide were used as described [25]. For drug treatments, three- to five-day-old seedlings were incubated in 1ml of liquid medium (half-strength MS medium) containing 50 µM brefeldin A (BFA), 33 µM wortmannin or 2 µM concanamycin A. The seedlings were incubated with inhibitors at room temperature for the indicated times before observation. Control treatments were performed with equal amounts of the DMSO. The following stock solutions were used: 50 mM BFA (Sigma-Aldrich) in DMSO; 33 mM wortmannin (Sigma-Aldrich) in DMSO; 2 mM concanamycin A (Sigma-Aldrich) in DMSO. Each treatment for confocal imaging was repeated at least three times with similar results.

### Confocal microscopy

For confocal analysis, seedlings mounted in half-strength MS liquid were analyzed with an upright Zeiss LSM 510 laser scanning microscope equipped with a META device. GFP or YPF was visualized by excitation with an Argon laser at 488 nm and detected with a 505- to 550-nm emission filter. For imaging of GFP/FM4-64, YFP/FM4-64, or GFP/chlorophyll, the signals were excited with an Argon laser at 488nm and detected with a spectral detector set BP 500-550 IR for green signal and LP 560 for red signal. Co-localization analyses were performed on F1 or F2 hybrid seedlings co-expressing GFP- and mCherry-tagged proteins under Zeiss LSM 5 LIVE. GFP and mCherry were excited with a 488-nm and 561-nm laser respectively (multitrack mode). The fluorescence emission was detected with spectral detector set BP 520-555 (GFP) and LP 575 (mCherry). To image propidium iodide and Calcofluor simultaneously by Zeiss LSM 5 LIVE, the parameter set was used as described [25]. Images were edited using ImageJ software (http://rsb.info.nih.gov/ij/) and Adobe Photoshop CS2.

## Supporting Information

Figure S1 **Characterization of wild type, *VAMP721* and *VAMP722* related mutants and rescued *vamp721vamp722* mutant plants.** (A) Wild type (Col-0), *vamp721*, *vamp722*, *vamp721^+/-^vamp722^-/-^*, *vamp721^-/-^ vamp722^+/-^* plants are shown from left to right in sequence. Note that all mutant plants are indistinguishable from the wild type. Bars  =  1 cm. (B) Wild-type and *vamp721vamp722* double mutant seedlings isolated from *vamp721^-/-^ vamp722^+/-^* plants are shown. Bars  = 5 mm. (C) *pVAMP721::GFP-VAMP721* fusion rescued the lethal double homozygous mutant. Bars  =  5 mm. (D) PCR verification of *vamp721vamp722* seedlings and complemented double homozygous mutant plants. Lines 1, 2, 4, and 5 are the PCR results of wild type and double mutant using the left genomic primer (LP) plus right genomic primer (RP) of both genes, as indicated. Lines 3 and 6 detect the T-DNA insertions of the double mutant.
(TIF)

Figure S2 **GFP-VAMP721 and GFP-VAMP722 exhibit strong signals at the cross walls in the abaxial epidermis of cotyledons.** (A) and (B) Arrowheads in panels indicate strong GFP-VAMP721 (A) and GFP-VAMP722 signals (B) at the cross walls in the abaxial epidermis of developing cotyledons. Bars  =  20 µm.
(TIF)

Figure S3 **Colocalization between mCherry-VAMP721 (green) and GFP-KNOLLE (red) at the cell plate and postcytokinetic wall in root mitotic cells.** Arrowheads indicate the expanding cell plates and arrows indicate the postcytokinetic walls. Bars  =  10 µm.
(TIF)

Figure S4 **GFP-VAMP721 and GFP-VAMP722 accumulate at the plasma membrane and cytoplasmic endosomes.** (A) and (B) Root tip cells expressing GFP-VAMP721 (A) and GFP-VAMP722 (B) (each green) incubated with FM4-64 (red) for 6 min. Note that GFP-VAMP721 and GFP-VAMP722 apparently labeled the plasma membrane and cytoplasmic endosomes colocalized with FM4-64 staining. Bars  =  10 µm.
(TIF)

Figure S5 **Massive intracellular accumulation induced by ConcA treatment.** (A–F) Root tip cells expressing GFP-KNOLLE (A, B), GFP-VAMP721 (C, D), and GFP-VAMP722 (E, F) were treated with ConcA for 2 h and then stained with FM4-64. DMSO was used as the control. Note that ConcA affects the morphology of GFP-KNOLLE-, GFP-VAMP721-, and GFP-VAMP722-labeled organelles. Bars  =  5 µm.
(TIF)

Figure S6 **The heterozygous double mutants show normal cytokinesis as observed in wild-type seedlings.** (A) and (B) Developing cotyledons of *vamp721^+/-^vamp722^-/-^* plants (A) and *vamp721^-/-^ vamp722^+/-^* plants (B) stained with propidium iodide displayed normal cytokinesis as observed in wild-type plants. Bars  =  20 µm. (C) and (D) *vamp721^+/-^vamp722^-/-^* plants (C) and *vamp721^-/-^ vamp722^+/-^* plants (D) did not show any cytokinetic defects in root tip cells stained with propidium iodide (red) and Calcofluor (green) simultaneously. Bars  =  10 µm.
(TIF)

Table S1 Primers used for constructs, T-DNA detection, and RT-PCR in this study.
(DOC)

Table S2 Quantification of cytokinetic phenotypes in wild-type, v*amp721vamp722* and complemented double mutant seedlings. The cytokinesis of root cells in wild type, v*amp721vamp722* and complemented double mutant seedlings was characterized by staining the cell walls and nucleus with Calcoflour and propidium iodide. The cells with one nucleus, two nuclei or incomplete cell walls (cell wall stubs or ruptured cell walls) were counted respectively. Total number of cells of a given genotype is indicated at right column.
(DOC)

Table S3 Quantification of cell plate-formation phenotypes in control, v*amp721vamp722* and complemented double mutant seedlings. The cell plate formation was monitored by GFP signals together with FM4-64 staining in cytokinetic root tip cells of GFP-KNOLLE transgenic lines used as the control, v*amp721vamp722* seedlings expressing this cell plate marker and complemented double mutant showing cell plate-labeling by GFP-VAMP721. The phenotypes of cell plate formation were designated defective assembly when cell plates with irregular direction and/or thickness could be seen, asymmetric assembly if a cell plate at either side of cell wall occurred, which usually results in cell wall stubs, and symmetric or complete expansion if the cell plate symmetrically expanded or attached to the parental membrane. Total number of cells of a given genotype is indicated at right column.
(DOC)
