# Supplementary figures and images for: Arabidopsis R-SNARE Proteins VAMP721 and VAMP722 Are Required for Cell Plate Formation
Source: PLoS One. 2011 Oct 11;6(10):e26129. doi: 10.1371/journal.pone.0026129 (PMC3191180; doi:10.1371/journal.pone.0026129)

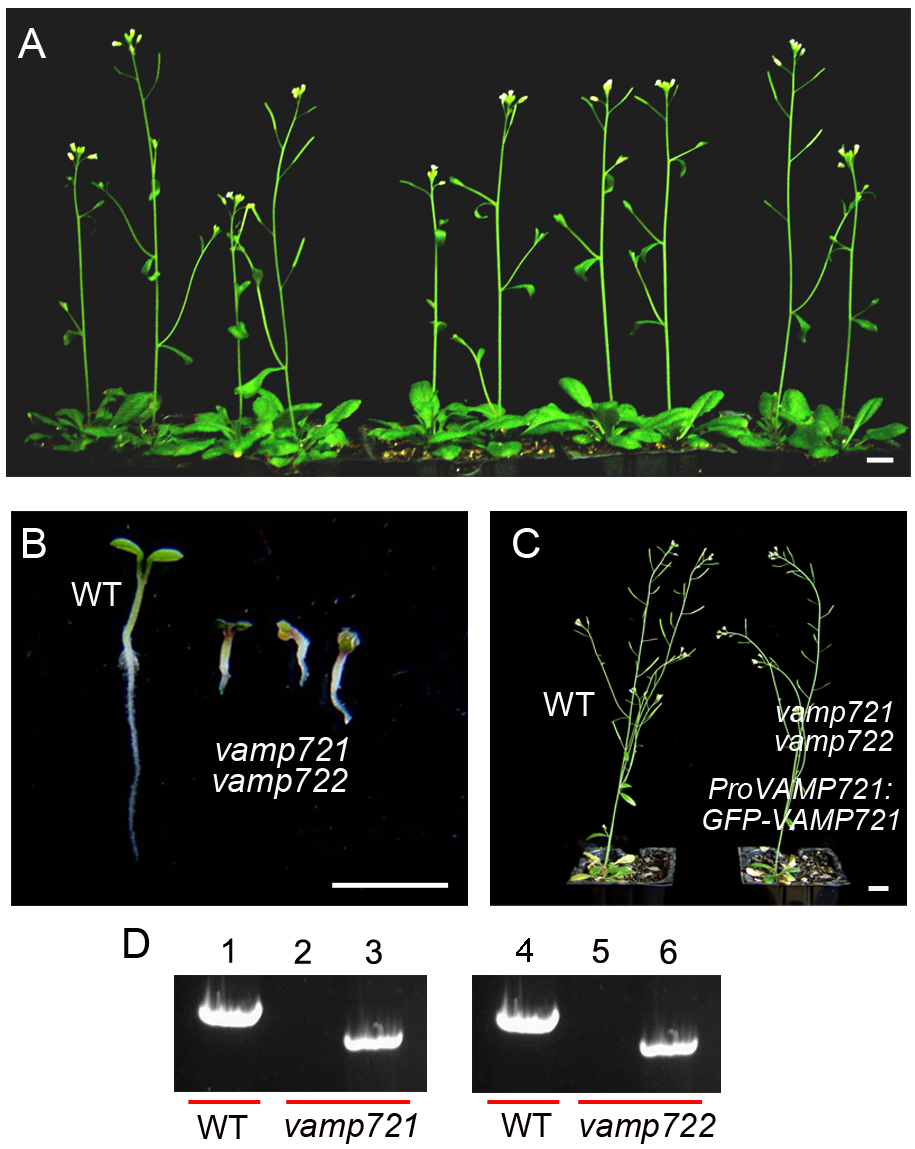

Supplement: Figure S1 — Characterization of wild type, VAMP721 and VAMP722 related mutants and rescued vamp721vamp722 mutant plants. (A) Wild type (Col-0), vamp721, vamp722, vamp721+/-vamp722-/-, vamp721-/- vamp722+/- plants are shown from left to right in sequence. Note that all mutant plants are indistinguishable from the wild type. Bars = 1 cm. (B) Wild-type and vamp721vamp722 double mutant seedlings isolated from vamp721-/- vamp722+/- plants are shown. Bars = 5 mm. (C) pVAMP721::GFP-VAMP721 fusion rescued the lethal double homozygous mutant. Bars = 5 mm. (D) PCR verification of vamp721vamp722 seedlings and complemented double homozygous mutant plants. Lines 1, 2, 4, and 5 are the PCR results of wild type and double mutant using the left genomic primer (LP) plus right genomic primer (RP) of both genes, as indicated. Lines 3 and 6 detect the T-DNA insertions of the double mutant. (TIF) [file pone.0026129.s001.tif]

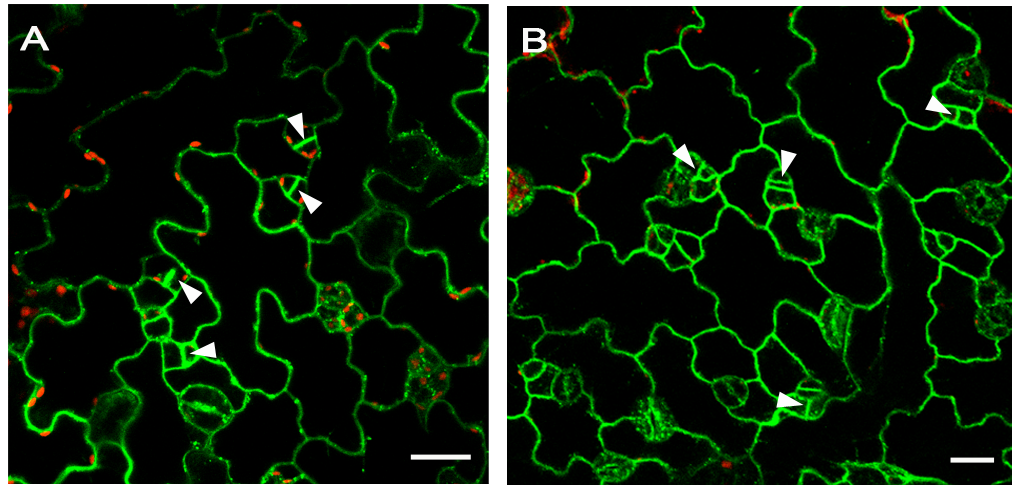

Supplement: Figure S2 — GFP-VAMP721 and GFP-VAMP722 exhibit strong signals at the cross walls in the abaxial epidermis of cotyledons. (A) and (B) Arrowheads in panels indicate strong GFP-VAMP721 (A) and GFP-VAMP722 signals (B) at the cross walls in the abaxial epidermis of developing cotyledons. Bars = 20 µm. (TIF) [file pone.0026129.s002.tif]

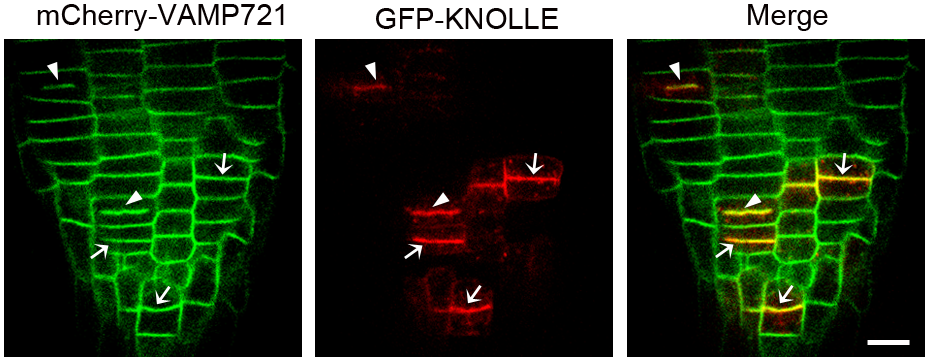

Supplement: Figure S3 — Colocalization between mCherry-VAMP721 (green) and GFP-KNOLLE (red) at the cell plate and postcytokinetic wall in root mitotic cells. Arrowheads indicate the expanding cell plates and arrows indicate the postcytokinetic walls. Bars = 10 µm. (TIF) [file pone.0026129.s003.tif]

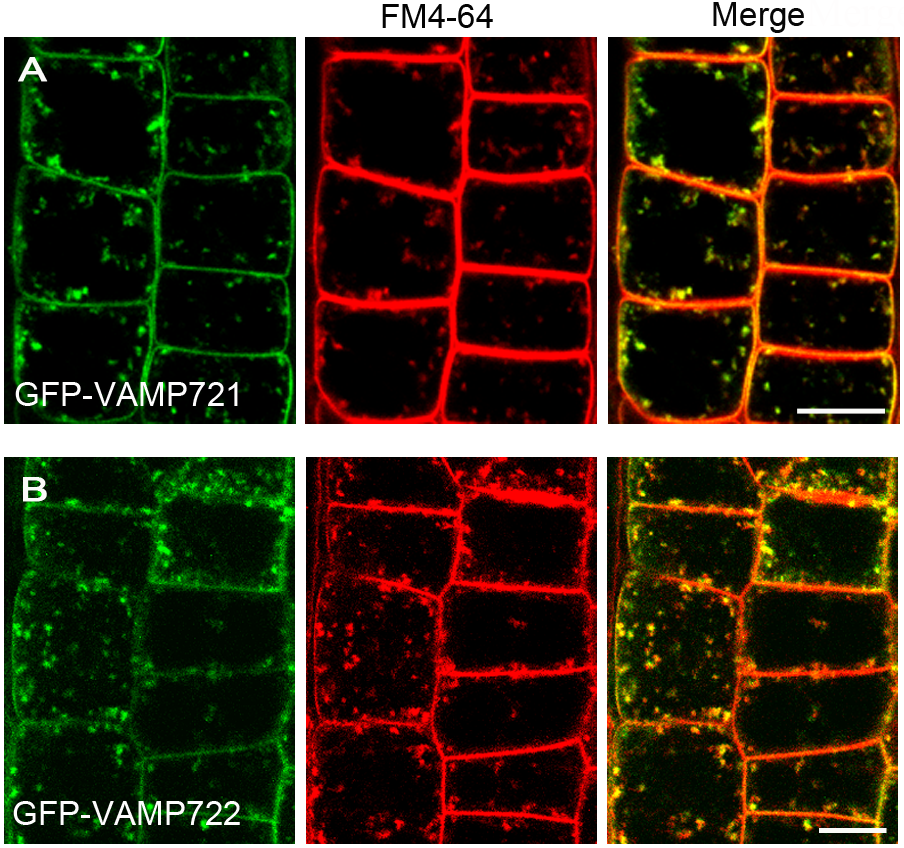

Supplement: Figure S4 — GFP-VAMP721 and GFP-VAMP722 accumulate at the plasma membrane and cytoplasmic endosomes. (A) and (B) Root tip cells expressing GFP-VAMP721 (A) and GFP-VAMP722 (B) (each green) incubated with FM4-64 (red) for 6 min. Note that GFP-VAMP721 and GFP-VAMP722 apparently labeled the plasma membrane and cytoplasmic endosomes colocalized with FM4-64 staining. Bars = 10 µm. (TIF) [file pone.0026129.s004.tif]

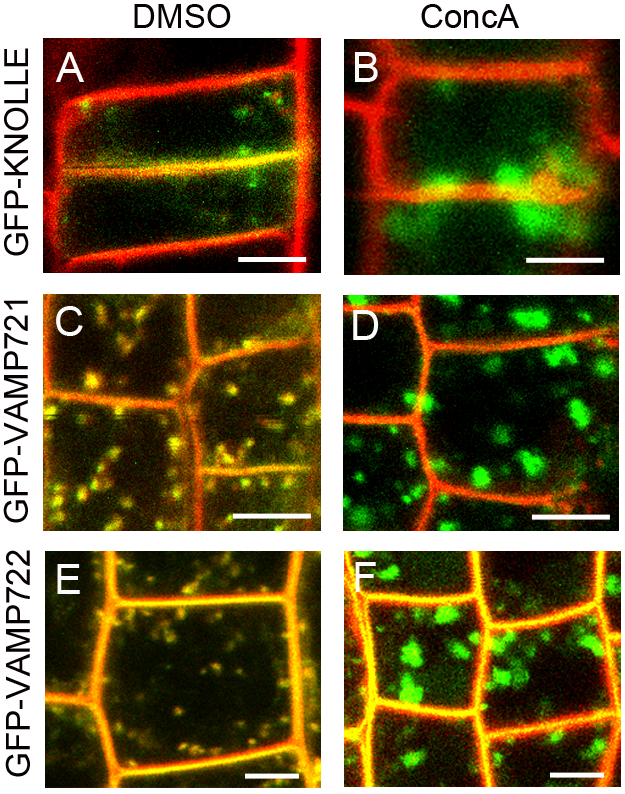

Supplement: Figure S5 — Massive intracellular accumulation induced by ConcA treatment. (A–F) Root tip cells expressing GFP-KNOLLE (A, B), GFP-VAMP721 (C, D), and GFP-VAMP722 (E, F) were treated with ConcA for 2 h and then stained with FM4-64. DMSO was used as the control. Note that ConcA affects the morphology of GFP-KNOLLE-, GFP-VAMP721-, and GFP-VAMP722-labeled organelles. Bars = 5 µm. (TIF) [file pone.0026129.s005.tif]

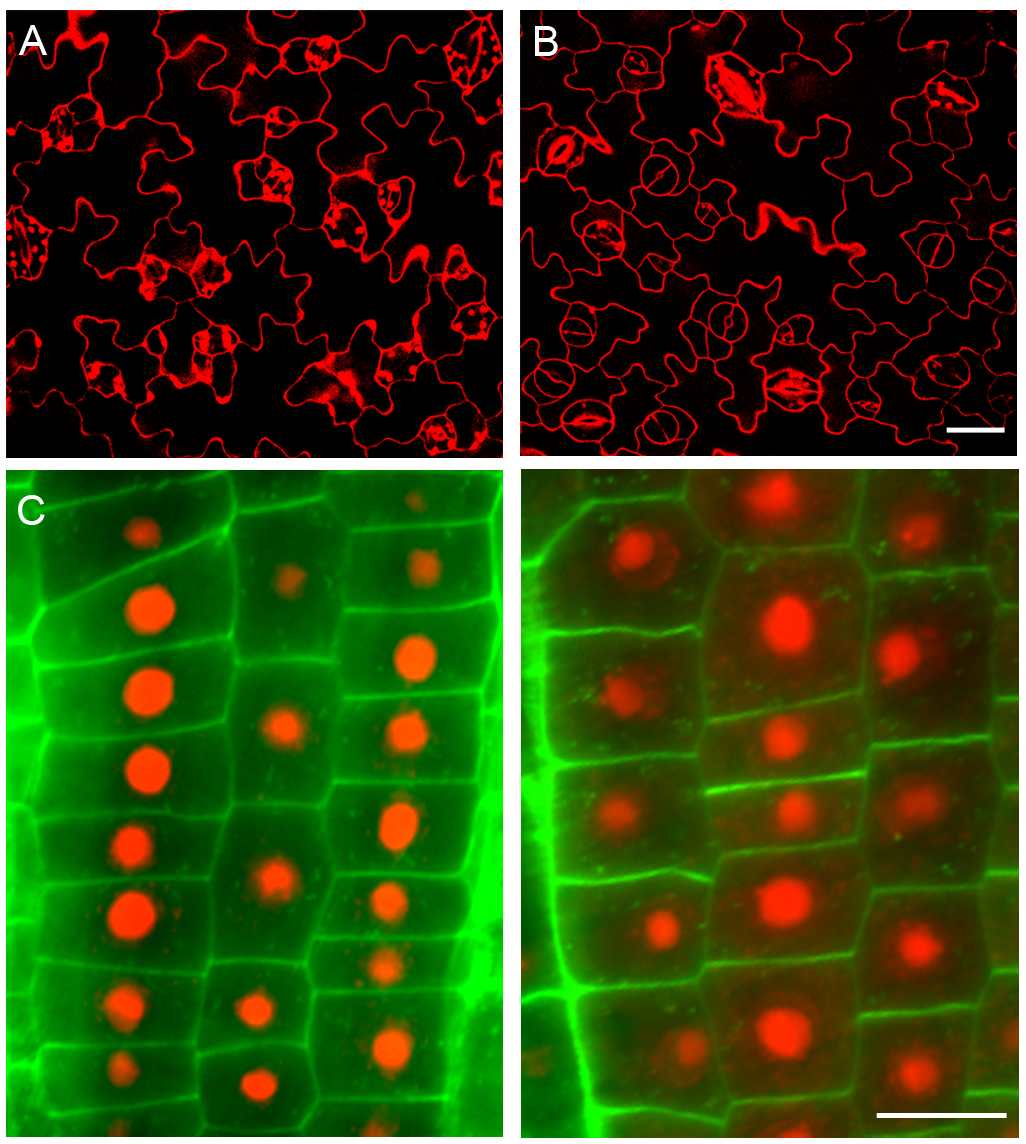

Supplement: Figure S6 — The heterozygous double mutants show normal cytokinesis as observed in wild-type seedlings. (A) and (B) Developing cotyledons of vamp721+/-vamp722-/- plants (A) and vamp721-/- vamp722+/- plants (B) stained with propidium iodide displayed normal cytokinesis as observed in wild-type plants. Bars = 20 µm. (C) and (D) vamp721+/-vamp722-/- plants (C) and vamp721-/- vamp722+/- plants (D) did not show any cytokinetic defects in root tip cells stained with propidium iodide (red) and Calcofluor (green) simultaneously. Bars = 10 µm. (TIF) [file pone.0026129.s006.tif]
