# Supplementary material for: Arabidopsis R-SNARE Proteins VAMP721 and VAMP722 Are Required for Cell Plate Formation
Source: PLoS One. 2011 Oct 11;6(10):e26129. doi: 10.1371/journal.pone.0026129 (PMC3191180; doi:10.1371/journal.pone.0026129)
Supplement: Table S1 — Primers used for constructs, T-DNA detection, and RT-PCR in this study. (DOC) [file pone.0026129.s007.doc]

**Table S1**

| Application | Sequence (5’-3’) (forward/reverse) |
| --- | --- |
| Cloning of Pro*_VAMP721_* | TATAAGCTTGCTTTCCAAACATAAAAATCTTC  (Underline = HindIII site) |
|  | TTGTCGACTTTTTCTTTACCTTAAATCTC  (Underline = SalI site) |
| Cloning of Pro*_VAMP722_* | TATAAGCTTTACTGAGTGATCCTGAGGTCGAG  (Underline = HindIII site) |
|  | TTGTCGACTTTTTTTACTCAAAAACGACGCG  (Unerline = SalI site) |
| Cloning of *VAMP721* | TAACTAGTATGGCGCAACAATCGTTGA  (Underline = SpeI site) |
|  | TAGCGGCCGCTTAACACTTAAACCCATGG  (Underline = NotlI site) |
| Cloning of *VAMP722* | TAACTAGTATGGCGCAACAATCGTTGA  (Underline = SpeI site) |
|  | TTGCGGCCGCTTATTTACCGCAGTTGAATC  (Underline = NotlI site) |
| Cloning of *mCherry* | TAGTCGACCGCCACCATGGTGAGCAAG  (Underline = SalI site) |
|  | ATACTAGTAGCTCCTCCTCCTCCCTTGTA  CAGCTCGTCCATGCCG  (Underline = SpeI site) |
| *vamp721* T-DNA insertion | SALK-LBb1  GCGTGGACCGCTTGCTGCAACTCTC |
|  | SALK037273-RP  ATTGAGGACAGAAAGGGTCAGATTC |
|  | SALK037273-LP  CCCCCGTCCATTAAGAATTAAG |
| *vamp722* T-DNA insertion | SALK-LBb1  GCGTGGACCGCTTGCTGCAACTCTC |
|  | SALK119149-RP  AACTATGCCCATGAATCATAGACCC |
|  | SALK119149-LP  CTCTGAGATCGGTCCCGTAAAATCGG |
| RT-PCR | *TUA3*f  GGACAAGCTGGGATCCAGG |
|  | *TUA3*r  CGTCTCCACCTTCAGCACC |
| RT-PCR | *VAMP721*f  ATGGCGCAAC AATCGTTGATC |
|  | *VAMP721*r  CACCACAATGCAATCTTCAGTC |
| RT-PCR | *VAMP722*f  GAAAGGCTGCAACTGCTCAA |
|  | *VAMP722*r  GCAGATTGTCGAACATGCATAC |
