# Supplementary material for: Arabidopsis R-SNARE Proteins VAMP721 and VAMP722 Are Required for Cell Plate Formation
Source: PLoS One. 2011 Oct 11;6(10):e26129. doi: 10.1371/journal.pone.0026129 (PMC3191180; doi:10.1371/journal.pone.0026129)
Supplement: Table S2 — Quantification of cytokinetic phenotypes in wild-type, vamp721vamp722 and complemented double mutant seedlings. The cytokinesis of root cells in wild type, vamp721vamp722 and complemented double mutant seedlings was characterized by staining the cell walls and nucleus with Calcoflour and propidium iodide. The cells with one nucleus, two nuclei or incomplete cell walls (cell wall stubs or ruptured cell walls) were counted respectively. Total number of cells of a given genotype is indicated at right column. (DOC) [file pone.0026129.s008.doc]

**Table S2**

| Cytokinesis (%) | | | | |
| --- | --- | --- | --- | --- |
|  | One nucleus cells | Binucleate cells | Incomplete cell walls | Total cells (n) |
| Wild-type | 97.8 | 1.3 | 0.9 | 234 |
| v*amp721vamp722* | 46.8 | 34.2 | 19.0 | 184 |
| Complemented double mutant | 96.5 | 2.2 | 1.3 | 228 |
