# Supplementary material for: Arabidopsis R-SNARE Proteins VAMP721 and VAMP722 Are Required for Cell Plate Formation
Source: PLoS One. 2011 Oct 11;6(10):e26129. doi: 10.1371/journal.pone.0026129 (PMC3191180; doi:10.1371/journal.pone.0026129)
Supplement: Table S3 — Quantification of cell plate-formation phenotypes in control, vamp721vamp722 and complemented double mutant seedlings. The cell plate formation was monitored by GFP signals together with FM4-64 staining in cytokinetic root tip cells of GFP-KNOLLE transgenic lines used as the control, vamp721vamp722 seedlings expressing this cell plate marker and complemented double mutant showing cell plate-labeling by GFP-VAMP721. The phenotypes of cell plate formation were designated defective assembly when cell plates with irregular direction and/or thickness could be seen, asymmetric assembly if a cell plate at either side of cell wall occurred, which usually results in cell wall stubs, and symmetric or complete expansion if the cell plate symmetrically expanded or attached to the parental membrane. Total number of cells of a given genotype is indicated at right column. (DOC) [file pone.0026129.s009.doc]

**Table S3**

| Phenotypes of Cell plate formation (%) | | | | |
| --- | --- | --- | --- | --- |
|  | Defective  assembly | Asymmetric  expansion | Symmetric**/**complete expansion | Total cells (n) |
| Control | 0.8 | 2.3 | 96.9 | 132 |
| v*amp721vamp722* | 41.8 | 10.9 | 47.3 | 110 |
| Complemented double mutant | 1.9 | 3.8 | 94.3 | 104 |
